# 

**Published:** 1895-07

**Authors:** 


### CONTENTS.

ORIGINAL COMMUNICATIONS—

LAPAROTOMY DURING PREGNANCY. By Virgil O. Hardon, M.D., Atlanta, Ga......... 257

ZOSTER. By Wm. S. Gottheil, New York........................................ 264

THE OPIUM CURSE AND A PREVENTION. By T. J. Happel, A.M., M.D., Trenton, Tenn. 270

RUDIMENTARY PREPARATION, COMPARED WITH HIGHER EDUCATION, FOR SPECIALTIES IN MEDICINE AND SURGERY. By J. McFadden Gaston, M.D., Atlanta, Ga.............. 278

SOCIETY REPORTS—

ATLANTA SOCIETY OF MEDICINE.............................................. 286

CORRESPONDENCE—

NEW YORK LETTER.......................................................... 291
PUDENDAL HEMATOCELE...................................................... 293

EDITORIAL—

THE PASSING OF THE ANTITOXIN............................................. 295
ORIFICIAL SURGERY........................................................ 298

SELECTIONS AND ABSTRACTS—

EYE, EAR, NOSE, AND THROAT................................................ 299
GENERAL MEDICINE AND THERAPEUTICS......................................... 304

BOOK REVIEWS.............................................................. 313
MEDICAL ITEMS............................................................. 318
ADVERTISERS' INDEX TO ADVERTISEMENTS...................................... xii
MEDICAL ITEMS—CONTINUED.................................................... x, xi
CLASSIFIED INDEX TO ADVERTISEMENTS........................................xxxv

